# Covid-19 and the need for more history and philosophy of RNA

**DOI:** 10.1007/s40656-021-00391-w

**Published:** 2021-03-23

**Authors:** Stephan Guttinger

**Affiliations:** Centre for Philosophy of Natural and Social Science, London School of Economics, Lakatos Building, Houghton Street, London, WC2A 2AE UK

**Keywords:** RNA, Coronavirus, COVID-19

## Abstract

RNA is central to the COVID-19 pandemic—it shapes how the SARS Coronavirus 2 (SARS-CoV-2) behaves, and how researchers investigate and fight it. However, RNA has received relatively little attention in the history and philosophy of the life sciences. By analysing RNA biology in more detail, philosophers and historians of science could gain new and powerful tools to assess the current pandemic, and the biological sciences more generally.

The history and philosophy of the life sciences (HPLS) offers powerful resources for the analysis of, and response to, the COVID-19 pandemic.^1^ Reflections on the nature of organisms; on evolutionary theory; the history of vaccination; or the potential of animal model research (to name just a few examples) can all contribute to the analysis of the current pandemic and its consequences.

However, there are also key elements of the pandemic that to date have gained little attention in HPLS. One of these elements is ribonucleic acid (RNA). In what follows I will look at two reasons why RNA matters for the pandemic: its role in viral behaviour and its importance as a tool in biomedical research. Given the central role of RNA, developing a history and philosophy of this molecule will provide HPLS scholars with important tools to analyse the unfolding of the pandemic and science’s response to it. Such a history and philosophy of RNA will also enable scholars to build a more detailed understanding of the contemporary life sciences, as RNA is becoming a more central element in biological theory and practice (Darnell, 2011). It also aligns with recent calls for a more pluralist approach to understanding the molecularization and the structure of the life sciences (Grote et al., 2021).

## RNA and the behaviour of viruses

The first and probably most prominent reason why RNA matters for the COVID-19 pandemic can be found in the genomics of SARS-CoV-2: like many other viruses—and unlike plants, animals, or bacteria—the genome of this virus consists of an RNA molecule.^2^ The structural difference between RNA and DNA at the nucleotide level is miniscule (a single additional hydroxyl group (–OH) in RNA nucleotides). This small difference, however, has broader consequences at the level of the polynucleotide. It changes how RNA genomes are packaged, regulated, and replicated. These changes in turn shape how RNA-based replicators behave and how they respond to interventions.

At the heart of these differences is the fact that RNA, in contrast to DNA, can form intricate three-dimensional structures on its own, ranging from simple hairpins to more elaborate higher-order arrangements.^3^ In the case of viral genomes these structures can be restricted to particular areas [for instance the 5′ untranslated region (5′ UTR)] or they can be distributed across the genome as a whole. In the latter case they are referred to as ‘genome-scale ordered RNA structure’ or GORS (Simmonds et al., 2004)).

The three-dimensional folding of RNA genomes raises new methodological and theoretical challenges. The structures have been shown to have functional relevance for all stages of the viral life cycle and virologists now often talk of two layers of information that viral genomes carry, the sequence layer and the structural layer (Boerneke et al., 2019). The two layers are not always neatly aligned; GORS, for instance, seem to be conserved across viral genotypes even in cases where there is little conservation at the sequence level (Simmonds et al., 2020). Traditional methods such as sequence alignments can therefore be of limited help when scientists try to identify conserved (and potentially functional) features of viral genomes. Scientists are thus expanding their methodological and conceptual toolbox, using thermodynamic stability and chemical probing to identify or predict such ‘supracoding elements’ (Fernández-Sanlés et al., 2017; Boerneke et al., 2019; Rangan et al., 2020). These new approaches to the functional analysis of viral genomes pushes functional genomics in new directions. These shifts, which can also be observed in human genomics, bring up new theoretical tensions and methodological questions (Guttinger and Love forthcoming). Analysing these emerging issues also matters for the study of SARS-CoV-2, which has been shown to contain numerous conserved structural features in its genome (Rangan et al., 2020).

Another important feature of most RNA viruses is their high mutation rate. This high rate of change at the sequence level means that any viral population will consist of a broad spectrum of variants. This has led virologists to use terms such as “quasispecies” or “mutant cloud” (rather than traditional terms such as “species”) when they describe RNA viruses (Domingo & Perales, 2019).^4^

The use of new metaphors such as “mutant cloud” might seem innocuous at first, but it has important consequences for scientific practice and theory. When researchers speak of “clouds” they shift their focus from thinking in terms of distinct and well-defined viral particles to thinking about a more distributed and dynamic entity. Cloud members interact with each other and with the cloud’s environment. This interconnectedness is functionally relevant, as it can affect viral fitness (Domingo & Perales, 2019) or even features that are usually seen as “intrinsic” properties of RNA viruses, such as their mutation rate. In the traditional picture of viruses each virus simply has “its” mutation rate, depending on the type of (error-prone) RNA polymerase that it encodes in its genomic sequence. Recent research on the actual mutation rates of RNA viruses (qua mutant clouds), however, has shown that the mutation rate is a more complex feature of a population that is also set to a large degree by cellular processes (such as RNA editing) that interact with the viral cloud (Cuevas et al., 2015).

This highly dynamic and context-dependent nature of RNA viruses also seems to matter in the case of SARS-CoV-2. It has been shown that SARS-CoV-2 is a quasispecies with high sequence diversity and dynamics, even within single patients (Jary et al., 2020). At the same time, there are indications that this virus does not mutate as fast as other RNA viruses (Callaway, 2020) and that the quasispecies can be surprisingly stable when the virus is replicated in cultured cells (Chaudhry et al., 2020). This indicates that the dynamics of SARS-CoV-2 are co-determined by the system within which it exists, in line with what a cloud-approach to viruses would predict.

In order to get a better understanding of how SARS-CoV-2 spreads and develops, it will therefore be important to gain a better understanding of the concept of viral clouds and its methodological and theoretical implications. Developing a more detailed history and philosophy of RNA biology is likely to help address these challenges.

## RNA as a key tool to study and fight viruses

It is not just the biology of viruses that makes RNA an important topic for HPLS in the context of the current pandemic (and beyond). A second reason is that RNA has transformed how researchers study and intervene in biological systems. If HPLS can develop a more detailed understanding of these RNA-related transformations in research practice, it will also further our understanding of how scientists analyse and tackle the current pandemic.

Underlying the growing power of RNA as a research tool is again the fact that RNA polynucleotides can fold into three-dimensional structures. This opens a broader spectrum of interactions with both DNA and proteins, making RNA a unique structural glue: rather than serving as a simple messenger that connects DNA with the protein translation machinery – an important but limited role that RNA was demoted to for decades in biological theory – RNA now emerges as a structurally complex *guide* for a range of DNA- or RNA-modifying enzymes, targeting them to specific sites on cellular or viral genomes.

The discovery of these “non-coding” RNAs (so called because they don’t code for protein products) has re-shaped how scientists understand the working of cells, the development of organisms, and the dynamics of evolution. Over recent years, non-coding RNAs have been identified as key players in processes such as the regulation of gene expression, genome stability, carcinogenesis, or anti-viral defence (Maillard et al., 2019; Mattick, 2018).

These discoveries have also had important methodological implications for how researchers study SARS-CoV-2 and other biological systems. Adapting RNA as a tool in the laboratory has allowed researchers to quickly and cheaply “re-programme” enzyme complexes, targeting them to specific loci simply by changing the sequence of the non-coding guide RNA. This principle of programmable enzyme complexes is used for RNA interference (RNAi) and also for novel gene editing tools, such as CRISPR-Cas9.^5^

The idea of using RNA to re-program biological processes is also used in the development of novel vaccines (Pardi et al. 2018). Rather than injecting, for instance, an attenuated virus or parts of a virus particle, researchers now inject an RNA that codes for viral proteins, forcing the cell to produce viral elements which then (hopefully) trigger an immune response. This is also one of the leading technologies currently used to quickly produce vaccines against SARS-CoV-2.^6^

The clear impact that RNA has had on research practice suggests that the dynamics, the power, and the limitations of the life sciences cannot be understood without also taking into account RNA biology. At the same time, ever since RNA has emerged as a key player in biological systems researchers have been forced to develop new concepts and to re-think existing models of biological mechanisms (for instance models of viral evolution or genomic regulation). Tracking and understanding these changes in research practice and in biological theory will represent an important task for HPLS, not only to build a stronger response to the COVID-19 pandemic, but also to build a better understanding of the modern life sciences more generally.

## Data Availability

Not applicable. Not applicable.

## Footnotes

1 This note belongs to the Topical Collection “Seeing Clearly Through COVID-19: Current and future questions for the history and philosophy of the life sciences”, edited by G. Boniolo and L. Onaga.

2 Note that RNA viruses are not a fringe part of the viral universe. Almost every virus that has grabbed the headlines over recent decades runs on RNA. Examples include HIV, Influenza, Measles, Ebola, Zika, Nipah, West Nile, Yellow fever, Dengue, Hepatitis, Polio, and the coronaviruses behind SARS and MERS.

3 This is due to the additional hydroxyl group. Some RNA molecules can form structures that are complex enough to serve a similar function to enzymes, i.e., proteins that catalyse chemical reactions. These so-called “ribozymes” are thought to have played a key role in the early origin of life (Robertson & Joyce, 2012). The conformational possibilities of RNA also make it a key structural and functional component of ribosomes, an essential element of the protein translation machinery.

4 The term “quasispecies” can of course be applied to any self-replicating system, but it has gained particular importance in the context of RNA viruses.

5 The former allows researchers to silence individual genes in cellular or viral genomes, whereas the latter allows researchers to delete or re-write particular genomic sequences with single-nucleotide precision. For an analysis of the emergence of “small RNA biology” see (Veigl, 2021).

6 https://www.nih.gov/news-events/news-releases/phase-3-clinical-trial-investigational-vaccine-COVID-19-begins. Two of the vaccines that are currently in use (Pfizer/BioNTech and Moderna) are based on RNA.
